# Role of Respiratory Syncytial Virus in Pediatric Pneumonia

**DOI:** 10.3390/microorganisms8122048

**Published:** 2020-12-21

**Authors:** Sonia Bianchini, Ettore Silvestri, Alberto Argentiero, Valentina Fainardi, Giovanna Pisi, Susanna Esposito

**Affiliations:** 1 Department of Medicine and Surgery, University of Perugia, 06123 Perugia, Italy; Bianchini.sonia@outlook.it (S.B.); ettore.silvestri@studenti.unipr.it (E.S.); 2 Pediatric Unit, ASST Santi Carlo e Paolo, 20142 Milan, Italy; 3 Pediatric Clinic, Pietro Barilla Children’s Hospital, Department of Medicine and Surgery, University of Parma, 43126 Parma, Italy; aargentiero85@gmail.com (A.A.); valentina.fainardi@gmail.com (V.F.); gpisi@ao.pr.it (G.P.)

**Keywords:** antiviral therapy, pneumonia, respiratory virus, RSV, vaccine

## Abstract

Respiratory viral infections represent the leading cause of hospitalization in infants and young children worldwide and the second leading cause of infant mortality. Among these, Respiratory Syncytial Virus (RSV) represents the main cause of lower respiratory tract infections (LRTIs) in young children worldwide. RSV manifestation can range widely from mild upper respiratory infections to severe respiratory infections, mainly bronchiolitis and pneumonia, leading to hospitalization, serious complications (such as respiratory failure), and relevant sequalae in childhood and adulthood (wheezing, asthma, and hyperreactive airways). There are no specific clinical signs or symptoms that can distinguish RSV infection from other respiratory pathogens. New multiplex platforms offer the possibility to simultaneously identify different pathogens, including RSV, with an accuracy similar to that of single polymerase chain reaction (PCR) in the majority of cases. At present, the treatment of RSV infection relies on supportive therapy, mainly consisting of oxygen and hydration. Palivizumab is the only prophylactic method available for RSV infection. Advances in technology and scientific knowledge have led to the creation of different kinds of vaccines and drugs to treat RSV infection. Despite the good level of these studies, there are currently few registered strategies to prevent or treat RSV due to difficulties related to the unpredictable nature of the disease and to the specific target population.

## 1. Introduction

**Figure 1:**
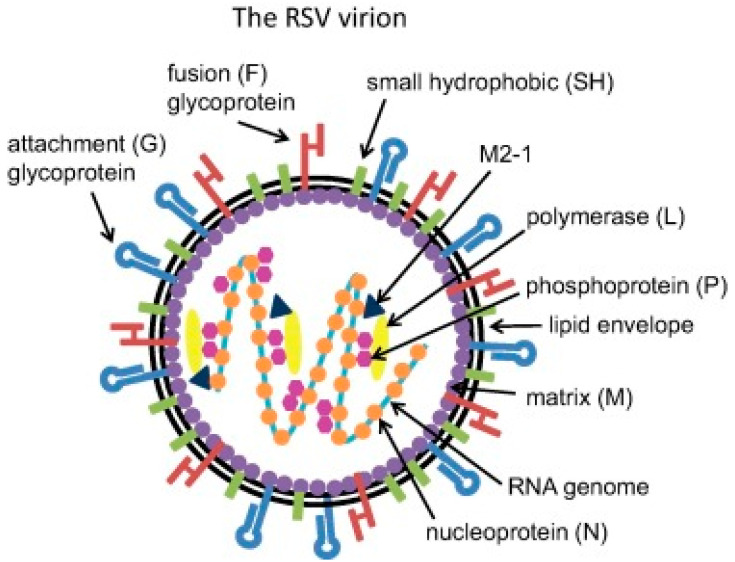
Structure of Respiratory Syncytial Virus (RSV).

Respiratory viral infections represent the leading cause of hospitalization in infants and young children worldwide and are the second leading cause of infant mortality [1,2]. Among these infections, Respiratory Syncytial Virus (RSV) represents the main cause of lower respiratory tract infections (LRTIs) in young children worldwide [3,4,5,6,7,8,9]. RSV is an enveloped, non-segmented, negative-strand RNA virus belonging to the *Paramyxoviridae* family [10]. RSV is divided into two antigenic subtypes, A and B, based on the reactivity of the F and G surface proteins to monoclonal antibodies [11]. The subtypes tend to circulate simultaneously within local epidemics, although subtype A tends to be more prevalent. Figure 1 shows the structure of RSV.

The global burden of RSV-associated acute LRTI is estimated at 33 million annually, resulting in more than 3 million hospitalizations and 59,600 in-hospital deaths in children aged under 5 years and 6.7% of all deaths in infants younger than one year old [12,13]. Furthermore, RSV-associated acute LRTIs account for 1.4 million hospitalizations and 27,300 in-hospital deaths in infants aged under 6 months [13,14,15,16]. Globally, RSV represents the major contributor to infant death in children worldwide [17]. An RSV wave starts in most countries in the Southern Hemisphere between March and June and in countries in the Northern Hemisphere between September and December [18,19,20,21,22]. A decrease in RSV activity was observed from August to October in the Southern Hemisphere and from February to May in the Northern Hemisphere [18,19,20,21,22].

RSV clinical manifestation ranges widely from mild upper respiratory infections to severe LRTIs, mainly bronchiolitis and pneumonia, leading to hospitalization, serious complications (such as respiratory failure) and relevant sequalae in childhood and adulthood (i.e., wheezing, asthma, and hyperreactive airways) [23,24,25,26,27].

Children in their first 2 years of life comprise the major risk group for RSV severe disease, with a peak in infants approximately 3 months old, after which the incidence gradually declines with age [28,29]. It has been speculated that almost all children aged < 2 years old experience at least one episode of RSV infection, and half of them are re-infected during their second or third year of life [30,31,32,33,34]. Risk factors for severe RSV infections are prematurity, low birth weight, male sex, bronchopulmonary dysplasia, congenital heart disease, immunodeficiency, cerebral palsy, and Down’s syndrome [35,36,37]. Moreover, children with so-called medical complexity (CMC), not only including subjects with previously cited specific chronic medical problems but also those with other potential lifelong conditions associated with medical fragility or relevant functional limitations necessitating care and/or require specific technological assistance, are at major risk of developing serious problems in cases of RSV infections [38,39,40,41]. Nevertheless, approximately 50–80% of emergency admissions related to RSV bronchiolitis occur in otherwise healthy term infants [41].

As RSV infections and their related problems represent a global burden worldwide, the World Health Organization (WHO) created a surveillance programme similar to that of influenza infection in 2017, with the aim of better understanding the incidence, seasonality, and regional patterns of this infection and the clinical aspects that lead to hospitalization. This programme has already entered phase II, which is planned to last until the end of 2021 [14,42]. Before this surveillance programme, Lam and colleagues gathered information about different respiratory viruses, including RSV, from 2010 to 2015 in 14 different countries, analysing the seasonal peaks in different parts of the world; the data revealed a notable pattern of synchrony for RSV (and influenza and parainfluenza viruses) incidence peaking globally, despite significant distances among the sites considered [43]. Moreover, collecting data from 27 countries between 2016 and 2017, the Obando-Pachebo group provided information that may allow the prediction of the beginning of RSV outbreaks worldwide [44].

This review aims to gather state-of-the-art information about RSV infection in children, specifically RSV pneumonia.

## 2. Incidence of Respiratory Syncytial Virus (RSV)

Globally, pneumonia is a major cause of paediatric infectious disease mortality and the greatest cause of death in children under 5 years old (with a 12.8% percentage of annual death beyond the neonatal period) [44,45,46,47]. Analysing an important sample of 36,500 paediatric pneumonia cases, the Tian group examined the meteorological conditions that influence RSV incidence: meteorological factors play an important role in the incidence of RSV, with the strongest correlation at the lowest temperature [48,49]. During the Pneumonia Aetiology Research for Child Health (PERCH) study, the authors reported a high prevalence of RSV infections in children living in high-burden, low-resource regions (of Africa and Asia), with 61% of children requiring hospital admission [50,51,52,53,54]. Moreover, the Global Approach to Biological Research, Infectious Disease and Epidemics in Low-income countries (GABRIEL) study reported an RSV incidence among pneumonia cases of 11.7%, placing it as the second most relevant pathogen, after influenza A, due to its high adjusted population attributable fraction (aPAF) of 18.2%; an inverse relationship between incidence of RSV pneumonia and age was also observed [55].

In 230 Australian children with pneumonia evaluated by the Bhuiyan group, RSV was detected in 20.2% (among the 44.4% amount of respiratory virus detectable globally), with a prevalence between May and November [56]. Nascimento and colleagues reported a lower incidence (approximately 15% for RSV-A and 12% for RSV-B) of RSV cases in patients with community-acquired pneumoniae (CAP), which was not as severe as hospitalization [57]. Additionally, the Khuri-Bulos group detected >95% viral infections during the winter period in their studied population, with a predominance of RSV, mainly in younger patients (approximately 20% in subjects younger than 6 months) and in those with pneumonia (approximately 35%) [58].

Romero-Espinoza and colleagues reported a high percentage of RSV, particularly serotype B, as the predominant species, along with Rhinovirus C, in subjects under 15 years old affected by pneumonia and/or asthma [59,60]. Moreover, Esposito et al. reported Italian circulating RSV types and serotypes (79.4% carried RSV-A and 20.6% RSV-B) among children presenting with influenza-like syndromes in five consecutive winter seasons [61].

RSV epidemics are seasonal, with peak infection occurring during late autumn/winter to early spring in the Northern Hemisphere [62,63,64].

## 3. Pathophysiology of Respiratory Syncytial Virus (RSV) Infection

**Figure 2:**
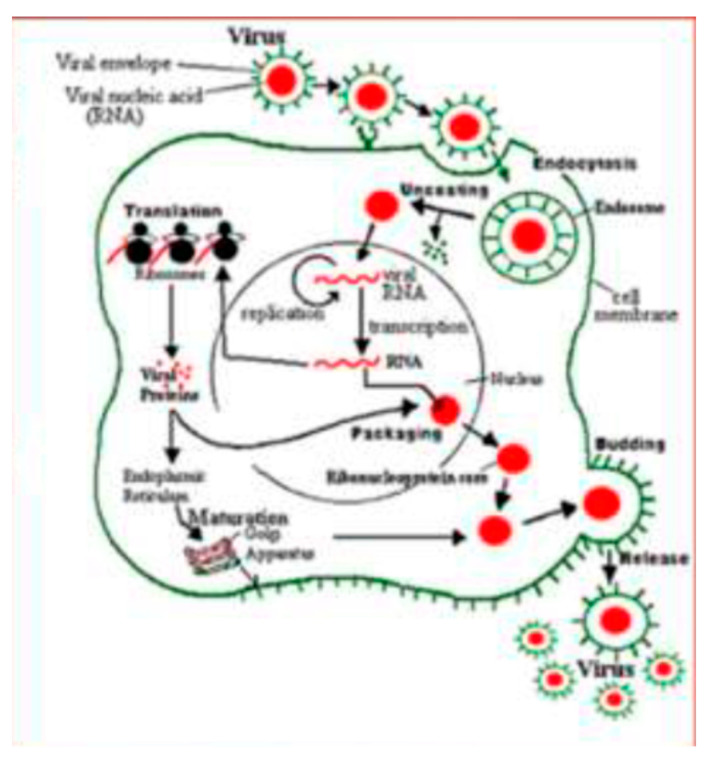
Binding and entry of Respiratory Syncytial Virus (RSV) into the host cell.

Many studies have attempted to explain the mechanism of entrance and the interaction of RSV with the human host (Figure 2) [65,66,67]. Lay and colleagues proposed a new model of pathogenesis. Firstly, RSV enters airway epithelial cells through interaction with the G protein; secondly, this attachment determines the binding of the RSV F protein to nucleolin (rich in cholesterol domain), which leads to activation of cytoskeleton and actin filament reorganization of the RSV envelope to introduce its content in the cytoplasm of the host cell [68]. 

RSV presents direct cytopathic activity, but signs and symptoms, which are virus dependent, are also due to the local host inflammatory response (innate and adaptative immune responses) created by its presence in airway epithelial cells [69,70,71,72,73]. Many in vitro and animal models have been employed to infer the pathological pathway of RSV, though its interaction with human hosts, and particularly with children, differs greatly from other models [74].

## 4. Clinical Manifestation of Respiratory Syncytial Virus (RSV)

RSV infections can range from mild, eventually even asymptomatic, to severe clinical infections, such as bronchiolitis and pneumonia. RSV can also cause severe pneumonia and acute respiratory distress syndrome (ARDS), which can lead to hospitalization and access to the intensive care unit (ICU) [75].

Focusing on pneumonia, which is the target of this review, and omitting better-known RSV bronchiolitis, subjects affected by RSV pneumonia usually present with the following features: fever, signs and symptoms of acute respiratory infections (cough, wheezing, tachypnoea), hypoxaemia and oxygen necessity, alteration of reactive C protein (CRP), and radiographic evidence (increased pulmonary texture or mottled shadows) [48,50,55,76,77].

In general, signs and symptoms appear after a short incubation period, as for the majority of viruses, of approximately 4 days [78]. The already cited prospective viral surveillance study of Khuri-Bulos and colleagues reported a significantly lower presence of fever but a high probability of cough, shortness of breath, and flaring in patients with RSV infection [58]. It has been observed that RSV-A causes more severe illness than RSV-B; in fact, the two serotypes elicit a different response in terms of cytokine and neutrophil production [74,79].

As mentioned before, there are some risk factors for RSV infection. Different studies have suggested the importance of any of these and of their association: prematurity, low birth weight, multiple birth, siblings, male sex, atopy, passive smoking and pollution, day care attendance, crowding, and malnutrition [80]. In a retrospective study, the Ravindranath group reported RSV as an important cause of paediatric acute respiratory distress syndrome (P-ARDS): analysing 161 RSV-positive patients admitted to the Paediatric Intensive Care Unit (PICU), the authors observed 20% P-ARDS among these subjects, who were younger than those with a milder presentation and had comorbidities [81].

RSV can cause symptomatic reinfection throughout life, even in subjects with healthy and mature immune systems. These infections can appear every two or three years, with different clinical manifestations according to age: the older the subject is, the milder is the infection; in fact, data highlight that in healthy adults, RSV only causes the common cold or limited upper respiratory tract infection [82].

Although there is partial protection against a specific RSV strain, consistent and durable protection is never achieved [83]. Some authors report evidence that the medium- and long-term alteration in mucosal innate responses may lead to protective innate memory and inflammatory signals that are essential for adaptative memory; indeed, the modulation of innate and adaptative responses may be responsible for the ability of RSV to re-infect [83,84].

RSV has been reported to modify airways and lead to problems later in childhood and even in adulthood. Acute wheezing illnesses, which are extremely common in young children, have a viral root in more than 60% of cases [85]. RSV has been associated with recurrent wheezing episodes and/or asthma [86,87], and the risk of wheezing is higher in patients who had more severe RSV infections [88]. The link between RSV infection and asthma has been well studied, though not completely understood. Rossi and colleagues proposed a role of inducer, rather than a trigger, for RSV in predisposing an individual towards asthma [69]. Other authors have suggested that asthma-related genetic traits themselves might predispose a person with RSV infection towards severe disease [68,89,90,91].

## 5. Diagnosis of Respiratory Syncytial Virus (RSV)

There are no specific clinical signs or symptoms that can distinguish RSV infection from other respiratory pathogens. However, identifying the aetiology of infection can enhance antimicrobial stewardship and reduce individual antibiotic-related complications [92,93]. The possibility of using technological point-of-care tests appears extremely important in terms of cost savings and even time used to organize where to place patients, isolated or not isolated [94].

Before the advent of novel rapid molecular diagnostic assays, the following were strategies available for detecting RSV: (1) cell culture (which has historically been considered the gold standard before the development of other techniques); (2) reverse transcriptase polymerase chain reaction (RT-PCR), which has recently been considered the new gold standard due to its excellent sensitivity and specificity and rapid turnaround time; (3) direct immunofluorescence assays (DFAs); and (4) rapid antigen detection tests (RADTs). RADTs are further divided into: (a) traditional antigen detection tests, such as immunochromatographic tests, enzyme immunoassays, and optical immunoassays, which required an operator to interpret the presence/absence of a positive signal, leading to variability; and (b) newer automated immunoassays [93].

Some authors have reported a relevant difference in RSV rapid antigen test sensitivity according to the age of the patient: the younger the patient is, the more accurate the test is. Starting from more than 80% in children younger than 6 months, sensitivity decreased to 60% in the 24–35-month-old group [95,96]. New multiplex platforms offer the possibility to simultaneously identify different pathogens, with an accuracy similar to that of single PCR in the majority of cases [61,78] (Table 1). Once the virus in the patient sample is identified, clinicians must decide whether the virus is truly responsible for the disease. As the state of RSV asymptomatic carriage is relatively uncommon, when RSV is detected, it is likely to be the cause of the clinical manifestation [78]. Nonetheless, the reported indisputable positive effects of molecular tests have some limitations. Firstly, viral RNA can persist beyond the period of clinical significance, and thus tests can detect both viable and nonviable pathogens, creating difficulties in interpreting results. Secondly, the majority of the available tests produce positive or negative results, with no information about the cycle threshold. Moreover, low-complexity assays do not differentiate between RSV subtypes A and B and do not provide a quantitative result. The number of samples analysed together can also vary widely. Finally, for some less recent tests, it might take longer to obtain results [78,93,97,98].

One of the best biological samples on which to perform RSV analysis with molecular testing is a nasopharyngeal swab obtained by trained personnel [99]. Therefore, the majority of studies rely on this technique, though the Turi group reported the successful identification of urinary metabolites in patients with acute respiratory infections due to RSV [100].

## 6. Treatment of Respiratory Syncytial Virus (RSV) Infection

Currently, the treatment of RSV infection relies on supportive therapy, mainly consisting of oxygen and hydration [101,102]. RSV pneumonia and severe LRTI represent the condition for which a treatment may be necessary. However, there are two main important obstacles to the creation of specific therapeutic strategies for RSV. Firstly, the virus undergoes changes during replication, which allows it to escape antiviral therapies. Secondly, treatments should be targeted to a specific population of young children, as RSV infection in older children or adults can be milder or even asymptomatic [103]. Despite these difficulties, antiviral drugs against a specific target of the virus are currently under study, as follows: (1) nucleoside analogues; (2) RNA interference; (3) fusion inhibitors; and (4) immunoglobulins other than palivizumab [103,104,105]. Among the antivirals studied to date, ribavirin represents the only licensed drug for RSV treatment. Nonetheless, its use is extremely limited to selected cases due to its potential toxicity [106].

## 7. Respiratory Syncytial Virus (RSV) Prevention

RSV prevention strategies include passive and active immunization. The two major RSV antigens, the F and G proteins, are the only two proteins targeted by neutralizing antibodies [107,108]. At present, the only prophylactic available for RSV infection is palivizumab, a humanized monoclonal antibody directed against a conserved epitope on the RSV F fusion protein that is administered once monthly during the winter season in selected patients at high risk of RSV-related complications [103,109,110,111,112].

The Domachowske group reported their positive experience with another prophylactic strategy against RSV: MEDI8897, a recombinant monoclonal antibody against the RSV F protein that has shown greater neutralizing activity and a longer serum half-life than palivizumab when administered in a single fixed dose to preterm infants (born between 32 and 34 weeks), for whom palivizumab was not recommended [113].

With regard to the development of vaccines, there are problems due to the following reasons: the target population, which is not easy to test; difficulties of transport evidence derived from adult studies, in whom RSV disease is different; and the capacity of RSV to evade the immune response. [64,73,114,115]. The history of vaccines dates backs to the 1960s, when a formalin-inactivated alum-adjuvated RSV vaccine (FI-RSV) administered in young (<2 years old) RSV-seronegative children resulted in enhanced respiratory disease during a subsequent natural RSV infection [83,107,116,117]. Recent advances in understanding RSV F protein structure and instability as well as improvements in technology have provided new targets for vaccines. Currently, there are approximately 60 vaccine candidates, some of which are at a good clinical phase. They can be divided into the following classes: (1) live attenuated vaccines, (2) particle-based vaccines, (3) subunit-based vaccines, and (4) vector-based vaccines [73,118,119]. Among these candidate vaccines are also ones for maternal immunization, which have been driven by the increasing number of countries that currently adopt vaccination in pregnant women against influenza and pertussis [73,120,121,122]. Vaccination against RSV during pregnancy may present two main benefits: (1) to protect mothers against this infection and its potential adverse birth outcomes and (2) to increase transplacental RSV-specific antibodies to protect the infant in the first months of life [37]. The Shi group estimated that achieving protection of 80% due to maternal immunization and prophylaxis in the first 6 months of life may prevent up to 1.1 million hospitalizations and 22,000 in-hospital deaths globally due to RSV [15].

Another possibility in terms of both RSV infection prevention and treatment that is currently being explored is the focus on potential host factor targets, such as RSV receptors, co-receptors, intracellular adhesion molecule, Toll-like receptor 4, and nucleolin [106,123,124,125].

At present, we found 23 active clinical trials on RSV prophylaxis and treatment, 10 of which are now in the recruitment phase. Of these, 2 are observational, multicentre studies, 4 are evaluating new vaccines (three in phase 1 and one in phase 2), 1 involves an RSV F protein inhibitor administered orally (RV521), and 3 are related to monoclonal antibodies (two, in phase 1 and 2, of the already mentioned MDI8897, and one involving MK-1654 in phase 2) [126].

## 8. Conclusions

RSV represents an insidious burden, especially for younger children, with a high mortality rate and a high percentage of serious infection and sequelae. Much progress has been made in understanding its mode of entry and interaction with the human host and its ability to cause disease. Advances in technology and scientific knowledge have led to the development of different kinds of vaccines and drugs to treat RSV infection. Despite the good level of some studies (that are now at phase 3 of clinical trials), there are still few registered strategies to prevent or treat RSV due to difficulties related to the unpredictable nature of the disease and the specific target population. However, it is highly likely that in the near future new preventive and therapeutic strategies against RSV will appear in the market, reducing its clinical and socio-economic impact.

## Figures and Tables

**Table 1:** Diagnostic strategies for RSV detection.

Test	Turnaround Time	Sensitivity	Specificity	Note
Cell culture	Days	Low	Excellent	Traditional gold standard; long time for results
RT-PCR	Hours	Excellent	Excellent	New gold standard; expensive; needs expertise; positive results may not indicate active infection; possibility of multiplex tests
DFAs	<1 h	Low	Low	Needs expertise; rapid results
RADTs	Minutes	Low	Variable	Traditional tests depend on operator skills; newer platforms have better sensitivity/specificity
Novel rapid molecular diagnostic assays	Minutes–few hours	Excellent	Good	Variability; not detection of RSV types; qualitative but not quantitative results

RT-PCR = reverse transcriptase polymerase chain reaction; DFAs = direct immunofluorescence assays; RADTs = rapid antigen detection tests.
